# Multidimensional profiling depicts infiltrating immune cell heterogeneity in the tumor microenvironment of stage IA non‐small cell lung cancer

**DOI:** 10.1111/1759-7714.14329

**Published:** 2022-02-11

**Authors:** Jin Duan, Guoli Lv, Nanye Zhu, Xin Chen, Yang Shao, Yong Liu, Wei Zhao, Yunfei Shi

**Affiliations:** ^1^ Department of Thoracic Surgery the First Affiliated Hospital of Kunming Medical University Kunming China; ^2^ Geneseeq Research Institute, Nanjing Geneseeq Technology Inc. Nanjing China; ^3^ School of Public Health Nanjing Medical University Nanjing China

**Keywords:** immune cell infiltration, immunohistochemistry, next‐generation sequencing, non‐small cell lung cancer, tumor microenvironment

## Abstract

**Background:**

Emerging evidence has underscored infiltrating immune cells in the tumor microenvironment (TME) of non‐small cell lung cancer (NSCLC). Owing to screening programs, the prevalence of early‐stage NSCLC is growing, but its high recurrence risk and poor survival impose an increasing demand for further understanding the TME.

**Methods:**

Tissue and plasma samples from 33 resectable stage IA NSCLC patients were collected from the surgery and subject to histological and genomic analyses. The distribution of CD8+ T cells, tumor‐associated macrophages (TAMs, M1 polarization and M2 polarization), and natural killer (NK) cells (CD56dim and CD56bright) was analyzed. The impact of clinical characteristics and immunotherapy‐related biomarkers on immune cell infiltration were also investigated.

**Results:**

Using multiplex immunohistochemistry (mIHC), we found a significantly higher M1 polarization proportion of total TAMs in tumor parenchyma than in other tissues, while other immune cells remained stable. Patients under 50 showed higher infiltrating CD8+ T cell density and M1 ratio in tumor tissues. Tumors carrying *RAS‐MAPK* mutations were associated with significantly increased infiltration of CD8+ T cells. We also identified significantly higher infiltration of CD8+ T cells and enrichment of CD56bright NK cells in high tumor mutation burden (TMB) and high programmed cell death ligand 1 (PD‐L1) samples, respectively.

**Conclusions:**

Our study highlighted the heterogeneity and dynamics of infiltrating immune cells in stage IA NSCLC TME, featured by M1 TAM enrichment in tumor parenchyma. Age, driver mutation type, TMB, and PD‐L1 level were found to associate with immune cell infiltration in the TME, shedding light on immunotherapy development.

## Graphical abstract

This study investigated immune cell infiltration in the tumor microenvironment (TME) of stage IA non‐small cell lung cancer (NSCLC) patients using multiplex immunohistochemistry (mIHC). We found the enrichment of M1 polarization of tumor‐associated macrophages (TAMs) in tumor parenchyma. In addition, age, driver mutation type, tumor mutation burden (TMB), and the programmed cell death ligand 1 (PD‐L1) level also affected immune cell infiltration in the early‐stage NSCLC TME.

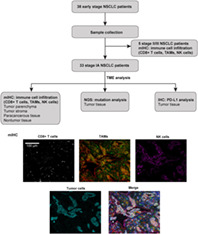

## INTRODUCTION

Lung cancer is the second most common cancer in the world, causing 25% of all cancer‐related deaths, while about 85% of lung cancer cases have been classified as non‐small cell lung cancer (NSCLC).^1^, ^2^ Although surgery is the standard primary treatment for early‐stage NSCLC, the overall 5‐year survival rate is still lower than 50%,^3^ and other primary treatments include radiation therapy and chemoradiation.^4^ Improving the cure rate of early‐stage NSCLC is currently one of the major challenges, whereas developing new therapeutic strategies, including immunotherapy, has gained growing attention. FDA‐approved immunotherapies are presently undergoing clinical trials for stage I lung cancer, including the programmed cell death ligand 1 (PD‐L1) inhibitor durvalumab as consolidation treatment with radiotherapy, as well as the PD‐1 inhibitor pembrolizumab.^5^, ^6^ As a wealth of emerging studies have discovered the importance of the tumor microenvironment (TME) in cancer, a better understanding will contribute to tumor immunology and immunotherapy.

The TME in solid tumors is a multidimensional system constituted by not only the tumor mass but also nonmalignant cells, such as fibroblasts, adipocytes, pericytes, vascular endothelial cells, and innate and adaptive immune cells.^7^, ^8^ Tumor‐infiltrating immune cells are the main players in the TME, consisting of T and B lymphocytes, natural killer (NK) cells, dendritic cells, macrophages, neutrophils, myeloid‐derived suppressor cells (MDSCs), etc.^7^ Immune cells have shown both antitumoral and protumoral roles at different stages of tumor progression. For instance, M1 tumor‐associated macrophages (TAMs) possess tumor‐antagonizing functions by producing proinflammatory cytokines and reactive oxygen/nitrogen species to kill tumor cells. In contrast, by producing anti‐inflammatory cytokines and suppressing immunosurveillance, M2 TAMs could promote tumors and appear to associate with poor survival of lung cancer patients.^9^, ^10^ Infiltrating immune cells can cause various effects on tumor progression, while their topological distribution and transition in lung cancer, as well as clinical implications, have not been fully elucidated. Meanwhile, tumor molecular and clinical factors are known to shape the tumor immune microenvironment and underlie the response to immunotherapy.^11^ A challenge for immunotherapy development is systematically understanding the nature and underlying mechanisms of infiltrating immune cells and their contributions to the TME.

Thus, immunotherapy could provide a promising alternative for early‐stage NSCLC patients with the risk of postsurgical recurrence, while their infiltrating immune cells in the TME have manifested a critical prognostic value.^12^ Here, we performed a multidimensional study combining histological and molecular approaches to investigate immune cell infiltration and profile the TME landscape of stage IA NSCLC patients. Our results could further contribute to biomarker discovery and immunotherapy development.

## METHODS

### Patient collection and assessment

We performed a retrospective study on 38 patients diagnosed with early‐stage, resectable lung adenocarcinoma between September 2020 and December 2020. Tissue samples were collected from the surgery. This study was approved by the Ethics Committee of the First Hospital of Kunming Medical University, Yunnan, China. Patient information was collected and assessed, including sex, age, tumor histology, stage, size, and smoking history.

The tissue samples from patients were fixed in a formalin solution and embedded in paraffin to prepare sections for hematoxylin and eosin (H&E) staining and immunohistochemistry (IHC) analysis. Cancer and noncancer tissue sections were evaluated by the investigators. Particularly, “paracancerous tissue” refers to the normal tissue within 0.5–1.0 cm from the tumor edge, and “nontumor tissue” refers to the normal tissue over 5.0 cm away from the tumor edge.

### Immunohistochemistry

We performed PD‐L1 staining with Dako‐brand anti‐PD‐L1 IHC 22C3 assay (Agilent Technologies). The specimen was then counterstained with hematoxylin and coverslipped. The PD‐L1 expression level was determined using the tumor proportion score (TPS), which is the number of tumor cells showing partial or complete membrane staining at any intensity divided by the total number of viable tumor cells multiplied by 100%. The value of TPS ≥1% was defined as PD‐L1‐positivity.^13^

### Multiplexed immunohistochemistry

Multiplex immunohistochemistry (mIHC) and multispectral imaging were used to identify immune cell subsets as described by Duan et al.^13^ In brief, mIHC was performed using PANO 7‐plex IHC kit (Panovue) according to the manufacturer's instructions. T cells were identified using the CD8 primary antibody (CD8α (C8/144B) Mouse mAb (IHC specific), CST70306S, Cell Signaling Technology). NK cells were identified using the CD56 primary antibody (NCAM1 (CD56) (123C3) Mouse mAb, CST3576S, Cell Signaling Technology) and were divided into CD56dim (weak staining) and CD56bright (bright staining) according to the intensity of membrane staining for the CD56 marker. TAMs were identified by CD68 antibody (CD68 recombinant rabbit monoclonal antibody RR654, BX00050, Biolynx) and HLA‐DR antibody (anti‐HLA‐DR antibody [TAL 1B5], ab20181, Abcam). They were further divided into type M1 (CD68+ and HLA‐DR+) and type M2 (CD68+ and HLA‐DR−). The anti‐panCK (recombinant anti‐pan cytokeratin antibody [C‐11], ab7753, Abcam) staining was used to stain tumor cells with malignant cytomorphology and sometimes confirmed by H&E staining slides. For mIHC, primary antibody incubations were sequentially applied, followed by horseradish peroxidase (HRP)‐conjugated secondary antibody incubation and tyramine signal amplification (TSA). The slides were microwave heated after each TSA application for epitope retrieval. The nuclear area of cells was stained by DAPI (4′,6‐diamidino‐2‐phenylindole) after the labeling of all the human antigens. Usually, 10–12 high‐powered fields (200×) from each tissue sample were randomly selected for analysis.

The Mantra System (Akoya Biosciences) was used to scan the slides by capturing fluorescent spectra at 20‐nm wavelength intervals from 420 to 720 nm with identical exposure times. We subsequently built the scans into a single stack image. Images of unstained and single‐stained sections were used to extract the spectrum of autofluorescence and fluorophores, respectively, from the tissue samples. The inForm image analysis software (Akoya Biosciences) was used to establish a spectral library required for multispectral unmixing from the autofluorescence extracted images, and then reconstitute images of the sections. We used AI‐assisted analysis to recognize marked cell subsets and determine their levels. The proportion of specific cell subtypes is calculated by the signal of colocalized DAPI‐cell subtype maker divided by the total DAPI‐positive signal. The ratios of TAM or NK cell subtypes were evaluated by dividing the number of target subtypes by the total number of TAMs or NK cells.

### Next‐generation sequencing (NGS) methods

DNA from the formalin‐fixed paraffin‐embedded (FFPE) samples was extracted using the QIAamp DNA FFPE Kit (Qiagen) following the manufacturer's instructions. DNA quality was assessed by spectrophotometry and quantified by Qubit 2.0 (Thermo Fisher).

Library preparation, NGS sequencing, and bioinformatic analysis have previously been reported.^14^ Briefly, 1 μg of fragmented genomic DNA was processed and subjected to hybridization‐based target enrichment of 425 cancer‐related genes with GeneseeqOne pan‐cancer gene panel (Geneseeq Technology Inc.). Libraries were amplified in KAPA HiFi HotStart Ready Mix (KAPA Biosystems) and quantified by qPCR using the KAPA library quantification kit (KAPA Biosystems). Target enriched libraries were sequenced on the HiSeq4000 platform (Illumina) with 2 × 150 bp pair‐end reads. The resulting sequences were then analyzed for base substitutions, insertions, deletions, copy number alterations, and gene fusions as described by Yang et al.^14^ The tumor mutation burden (TMB) was calculated based on the number of nonsilent somatic mutations per megabase coding region sequenced in the targeted panels.^15^

### Statistical analysis

For statistical analysis, a student's *t*‐test were performed with Graphpad Prism (version 9.0.0). All statistical tests were two‐sided, and a *p*‐value <0.05 indicated significant statistical differences (0.01 < *p* < 0.05: *; 0.001 < *p* < 0.01: **; *p* < 0.001: ***).

## RESULTS

### Patient overview

From the total 38 NSCLC patients, we diagnosed 33 with stage IA NSCLC cases that were analyzed throughout this study according to the AJCC TNM staging system. The other five were diagnosed with stage II or III NSCLC samples, and we only performed mIHC as the comparison control. The clinical characteristics of the 33 stage I patients, including age, sex, histological type, smoking history, tumor mutation burden, and PD‐L1 levels was summarized in Table 1. The clinical characteristics of stage II/III patients is listed in Table S1.

**TABLE 1:** Clinical characteristics of stage IA lung cancer patients

Characteristics	All patients (*N* = 33)
Median age, years (range)	49 (29–76)
Median tumor diameter, cm (range)	0.8 (0.3–3.0)
Sex, *n* (%)	
Male	10 (30.3%)
Female	23 (69.7%)
Histology, *n* (%)	
MIA	7 (21.2%)
ADC	26 (78.8%)
Stage, *n* (%)	
IA	33 (100.0%)
TNM, *n* (%)	
T1a	24 (72.7%)
T1b	4 (12.1%)
T1c	5 (15.2%)
Smoking, *n* (%)	
Current smoker or ever smoker	5 (15.2%)
Never smoker	28 (84.8%)
TMB (mut/Mb), *n* (%)	
≥10	2 (6.1%)
<10	31 (93.9%)
PD‐L1 level, *n* (%)	
≥1%	7 (21.2%)
<1%	26 (78.8%)

Abbreviations: ADC, invasive adenocarcinoma; MIA, minimally invasive adenocarcinoma; TMB, tumor mutation burden.

### Spatial distribution of infiltrating immune cells

**FIGURE 1:**
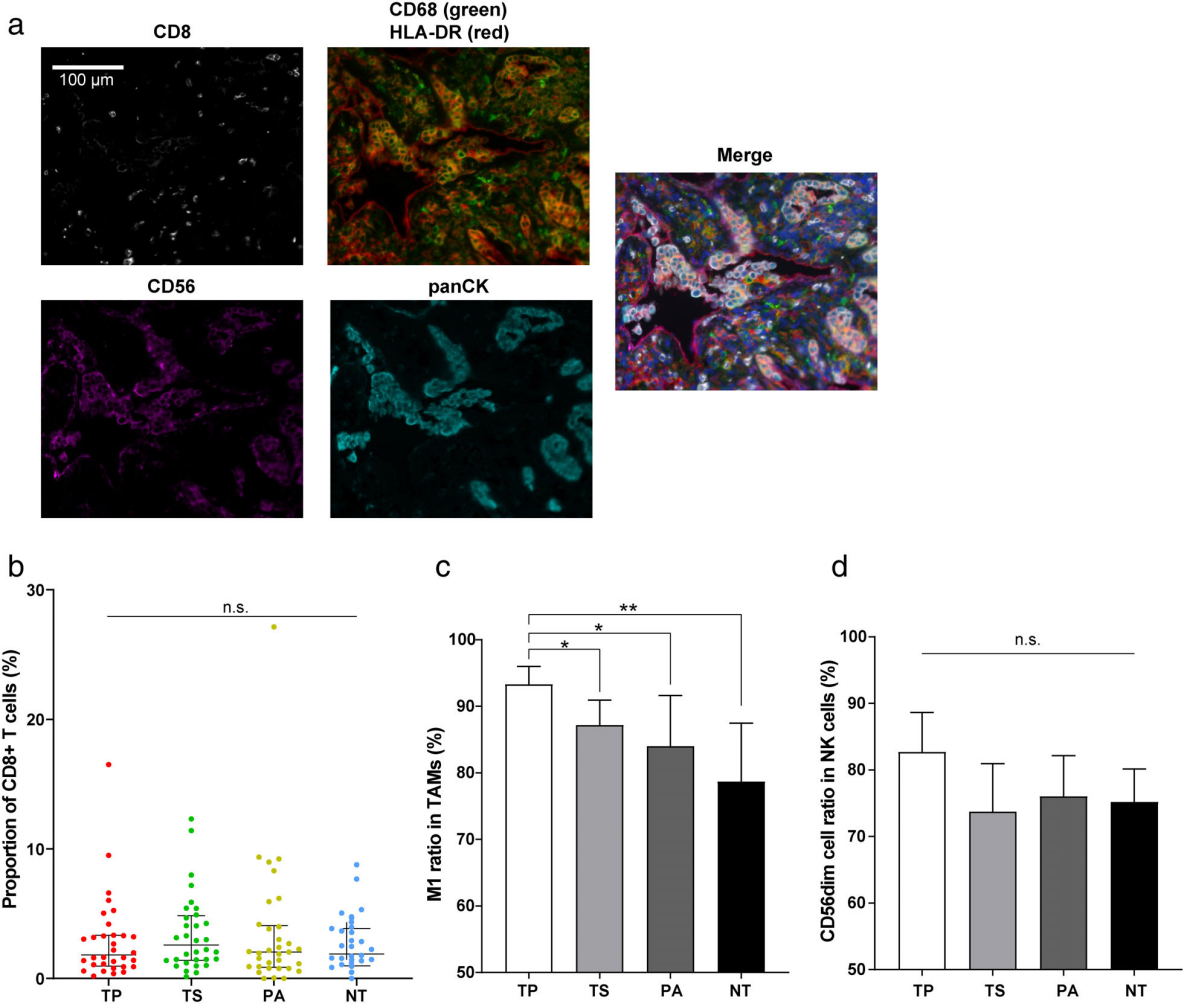
Spatial distribution of immune cells by mIHC imaging. (a) Representative micrographs of mIHC and multispectral imaging, at 200× magnification. CD8+: T cells; CD56+: NK cells; CD68+ HLA‐DR+: M1 TAM; CD68+ HLA‐DR‐: M2 TAM; panCK, tumor cells. DAPI nuclear staining is shown in blue in the merged micrograph. Scale bar indicates 100 μm. Distribution of (b) CD8+ T cells, (c) TAMs, and (d) NK cells were calculated in different tissue samples. All the scatter plots and bar graphs in this study indicate the corresponding median value and 95% confidence interval unless noted otherwise (mIHC, multiplexed immunohistochemistry; TAM, tumor‐associated macrophage; NK cells, natural killer cells; TP, tumor parenchyma; TS, tumor stroma; PA, paracancerous tissue; NT, nontumor tissue)

Using the mIHC technique (Figure 1a), we assessed the spatial distribution of infiltrating immune cells. In detail, we checked CD8+ T cells, CD68+ TAMs, and CD58+ NK cells in the tumor parenchyma (TP), stroma (TS), paracancerous tissue (PT), and nontumor tissue (NT). As shown in Figure 1b, the four different tissues of stage I lung cancer showed no statistical difference in the density of infiltrating CD8+ T cells. The proportions of CD68+ TAMs are also consistent across different tissues except that the tumor parenchyma is moderately higher than the nontumor region (*p*‐value = 0.04, Figure S1A). For CD56+ NK cells, their local density level in tumor parenchyma is significantly higher than other tissues (*p*‐value = 0.001, 0.006, and <0.001, Figure S1B). Due to the different functional roles of M1 and M2 TAMs in tumor immunity, we calculated the ratio of antitumoral M1 in total TAMs (M1 + M2) and assessed the functional difference of the TAM population represented by the relative M1 ratio in different tissues. Our analysis revealed a significantly increase of the relative M1 proportion in tumor parenchyma compared to other tissues (TP vs. TS, *p*‐value = 0.01; TP vs. PA, *p*‐value = 0.03; TP vs. NT, *p*‐value = 0.006, Figure 1c). We also compared subpopulations of CD56+ NK cells. The relative proportion of CD56dim NK cells is slightly higher in tumor parenchyma than in other locations but lacks statistical significance (Figure 1d).

Next, we compared the mIHC results in the tumors of 33 stage I patients and five stage II/III patients to evaluate the transition of infiltrating immune cells during NSCLC development (Figure S2). There is a significant decrease of the M1 proportion in the tumor parenchyma of stage II/III patients (Figure S2A). We also observed a slight increase of CD8+ T cell density in stage II/III but lacked statistical significance (Figure S2B), while essentially no change with NK cells (Figure S2C). Collectively, our mIHC results demonstrated the complexity and heterogeneity of filtrating immune cells in the TME of NSCLC across different locations and stages.

### Association between clinical characteristics and immune cell infiltration

**FIGURE 2:**
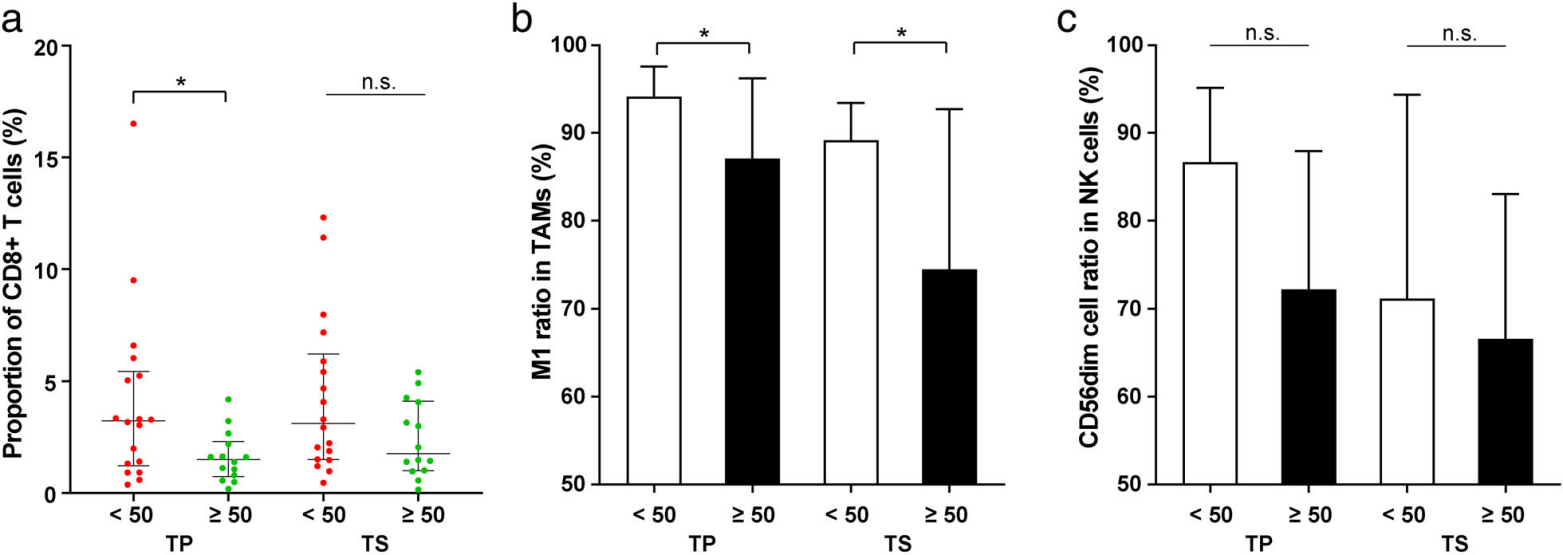
Abundance of (a) CD8+ T cells, (b) TAMs, and (c) NK cells in tumor parenchyma and stroma between patient age < 50 years and ≥ 50 years (TAM, tumor‐associated macrophage; NK cells, natural killer cells; TP, tumor parenchyma; TS, tumor stroma)

To explore clinical characteristics associated with the prevalence of infiltrating immune cells, we initially assessed the features, including sex, age, and disease histology (Table 1), and found age appeared to affect the TME in cancer tissues (Figure 2). Patients of age < 50 showed higher prevalence of CD8+ T cells in tumor parenchyma (*p*‐value = 0.04, Figure 2a), and higher ratios of M1 TAMs in both tumor parenchyma and stroma (*p*‐value = 0.05 and 0.04, respectively, Figure 2b). Our stage IA samples include seven minimally invasive adenocarcinomas (MIAs) and 26 invasive adenocarcinomas (ADCs). The comparison suggested an upward trend of CD8+ T cell infiltration in tumor parenchyma from MIA to ADC but lacked statistical significance (Figure S3).

**FIGURE 3:**
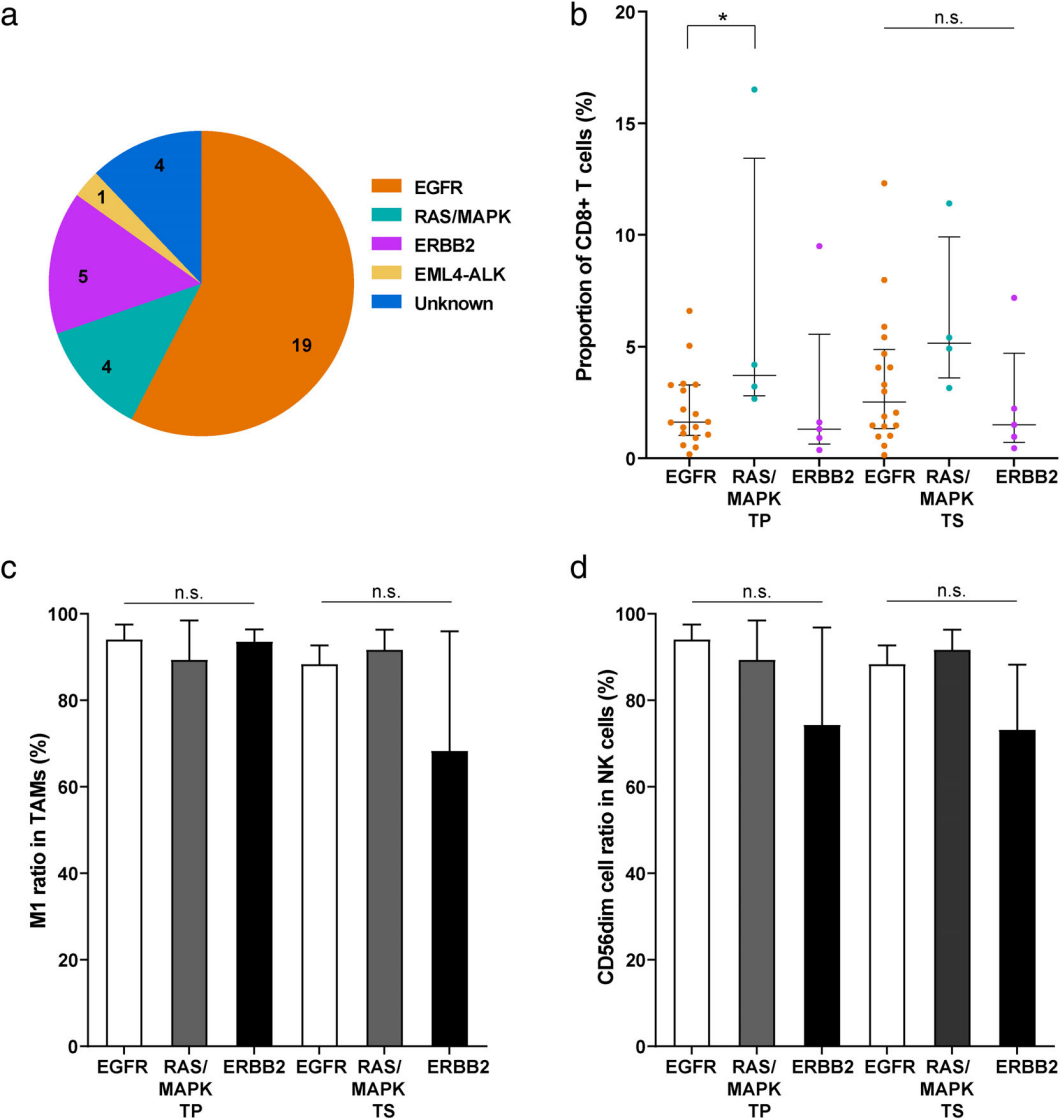
Abundance of infiltrating immune cells in tumor parenchyma and stroma among patients harboring different tumor driver mutations. (a) Summary of tumor driver mutations in the stage IA patients. The abundance of (b) CD8+ T cells, (c) TAMs, and (d) NK cells in patients carrying *EGFR*, *RAS‐MAPK*, and *ERBB2* driver mutations (TAM, tumor‐associated macrophage; NK cells: Nnatural killer cells; TP, tumor parenchyma; TS, tumor stroma)

**FIGURE 4:**
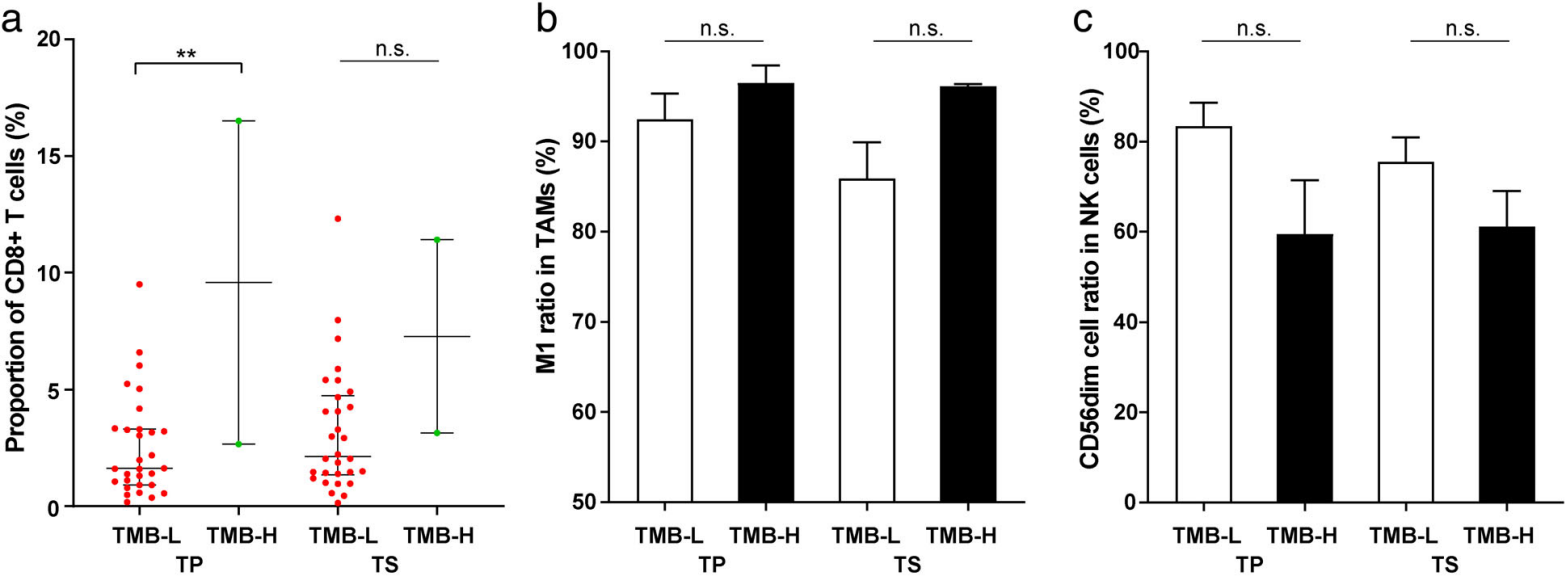
Abundance of (a) CD8+ T cells, (b) TAMs, and (c) NK cells in tumor parenchyma and stroma between TMB‐L (< 10 muts/Mb) and TMB‐H patients (≥ 10 muts/Mb) (TAM, tumor‐associated macrophage; NK cells, natural killer cells; TP, tumor parenchyma; TS, tumor stroma; TMB‐L, tumor mutation burden low; TMB‐H, tumor mutation burden high)

**FIGURE 5:**
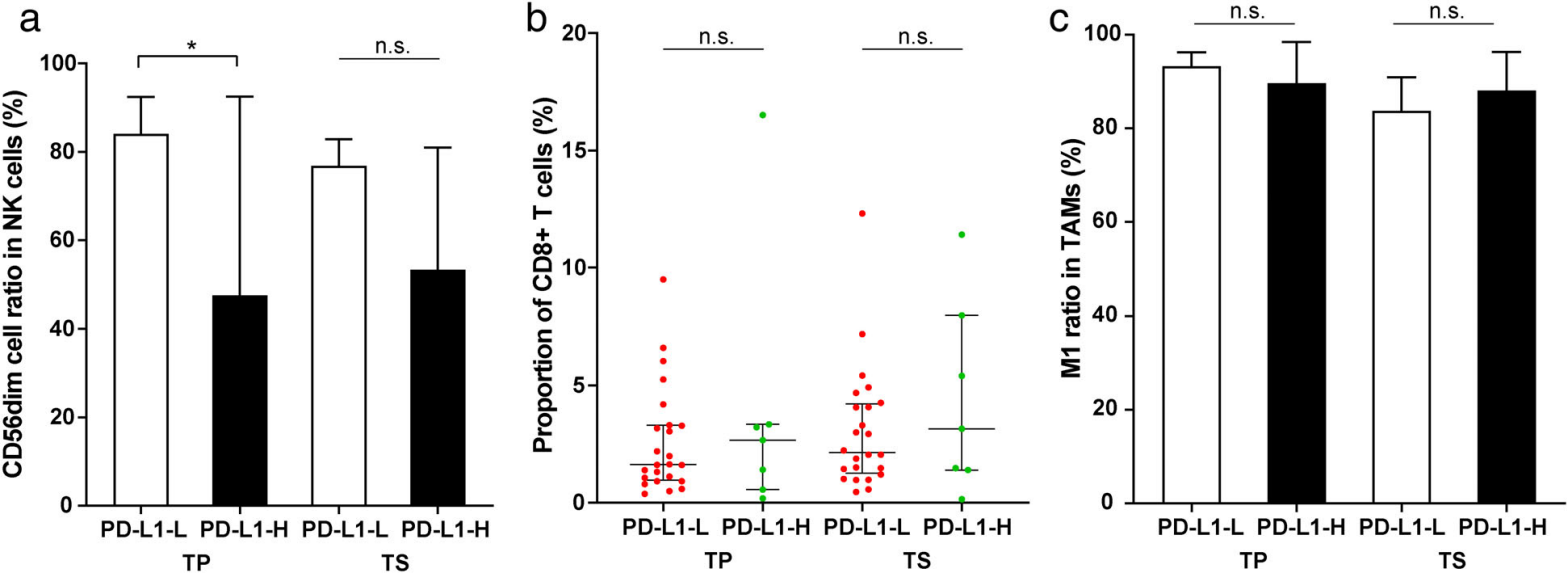
Abundance of (a) NK cells, (b) CD8+ T cells, and (c) TAMs in tumor parenchyma and stroma between PD‐L1‐L (< 1%) and PD‐L1‐H patients (≥ 1%) (TAM, tumor‐associated macrophage; NK cells, natural killer cells; TP, tumor parenchyma; TS, tumor stroma; PD‐L1‐L, programmed cell death ligand 1 low; PD‐L1‐H, programmed cell death ligand 1 high)

The known late‐stage lung cancer biomarkers related to immunotherapy, including driver mutations, PD‐L1, and TMB levels, were also evaluated for their effects on the stage IA NSCLC TME. We performed panel‐based next‐generation sequencing to identify mutations in cancer‐related genes of the stage IA patients (Figure 3a and S4). The most abundant driver mutation class in the patients was *EGFR* mutations (*n* = 19), followed by *ERBB2* (*n* = 5) and *RAS‐MAPK* (*n* = 4) mutations. Comparison of the three driver mutation classes revealed that tumors containing *RAS‐MAPK* mutations showed higher infiltration of CD8+ T cells than the two classes. In particular, the infiltrating CD8+ T cell levels are significantly different between *RAS‐MAPK* and *EGFR* groups in tumor parenchyma (*p*‐value = 0.01, Figure 3b). The difference in driver mutation type did not significantly alter the ratio of TAM or NK cell subtypes in the tumor tissue (Figure 3c and d). Meanwhile, *TP53* mutations are among the most common mutations in lung cancers and are considered relatively later molecular events during tumor progression.^16^ Four patients carried heterogenous *TP53* mutations (Patient 1: missense variant, c.526T>C[p.C176R]; Patient 2: missense variant, c.844C>T[p.R282W]; Patient 3: stop gain variant, c.772G>T[p.E258*]; Patient 4: splice acceptor site variant, c.920‐1G>A) in this study (Figure S4). They display loss of function and account for 12.1% (4/33) of stage IA patients here, consistent with the frequencies reported in other early‐stage lung cancer studies.^16^, ^17^, ^18^, ^19^ We compared the *TP53*‐mutated and *TP53‐*wild‐type patients and found no significant difference in immune cell infiltration (Figure S5). The infiltrating CD8+ T cells in tumor parenchyma appeared to be related to the TMB level, as TMB‐H (≥10 mutations per megabase, muts/Mb) samples exhibited a significantly higher prevalence of CD8+ T cells than TMB‐L (<10 muts/Mb) samples (*p*‐value = 0.002, Figure 4a). The relative ratios of M1 TAMs and CD56dim NK cells showed milder changes in cancer tissues between TMB‐L (low) and TMB‐H (high), but the differences are not significant (Figure 4b and c). We quantified the PD‐L1 expression by tumor proportion score (TPS) and chose 1% to distinguish PD‐L1‐H (high, ≥1%) and PD‐L1‐L (low, <1%). In the tumor tissues, the ratios of CD56dim NK cells were higher in PD‐L1‐L samples (Figure 5a), and the difference is statistically significant in tumor parenchyma (*p*‐value = 0.03). There was no significant difference in other infiltrating immune cells regarding the PD‐L1 level (Figure 5b and c).

## DISCUSSION

To our best knowledge, here, we present the first TME study profiling the distribution and potential functional changes of different infiltrating immune cells for stage IA NSCLC. Our study indicated that infiltrating immune cells exhibit heterogeneous distribution in the TME associated with particular clinical characteristics and immunotherapy biomarkers. The increasing prevalence of early‐stage NSCLC due to screening programs has posed a growing need to improve the treatment options. For instance, while a significant proportion of stage IA NSCLC patients can be cured with surgery, radiation therapy is a viable alternative for inoperable patients,^20^ but appears to display inferior survival than surgical resection.^21^ Combining immunotherapy and radiation therapy may work better than radiation therapy alone. Related ongoing clinical trials include testing the addition of atezolizumab to the usual radiation treatment for inoperable stage IA NSCLC patients.^22^ In addition, despite a lower mortality rate than those in the later stages of disease, surgically‐treated stage IA NSCLC patients still show varying survival and have a high risk of relapse, especially for high‐risk factor patients.^23^, ^24^ At the same time, adjuvant chemotherapy and radiation therapy are not recommended at this time.^25^ Hence, it is important to develop new therapeutic approaches to effectively treat these early‐stage patients and create a sustained systemic response. Multiple clinical trials are underway to explore the neoadjuvant and adjuvant use of immunotherapy for stage IA NSCLC.^6^, ^26^, ^27^ A better understanding of immune cell infiltration in the early‐stage TME would shed light on promoting immunotherapy.

In this study, a major finding is the heterogeneity of TAM subgroups in the early NSCLC TME. TAMs are critical components in the TME and highly plastic to adopt either protumor or antitumor features in response to tumor microenvironment stimuli.^28^ Previous studies have suggested the controversial roles of TAM density on cancer prognosis.^29^, ^30^, ^31^ We observed a higher density of total TAMs in tumor parenchyma than in other tissues in stage I NSCLC (Figure S1A). Furthermore, there is a significantly higher proportion of the M1 subgroup in tumor parenchyma, but it decreases as the disease progresses to stage II/III (Figure 1b and S2A). This finding is consistent with the decrease of M1 TAMs from stage I to stage III and the observed increase of M2 TAMs in stage II and III patients.^9^, ^32^ We also found that the M1 TAMs are relatively more abundant in the tumor tissues of younger patients (Figure 2b). M1 and M2 TAMs are two discrete activation stages of macrophage. The former inhibits angiogenesis and activates antitumoral immunity, and the latter facilitates tumor growth, invasion, and metastasis.^9^ Therefore, our results suggested a potentially functional transition of TAMs in the TME of NSCLC from relatively more tumor‐antagonizing to more tumor‐promoting during disease progression or aging (mean age: 50.9 in stage IA vs. 51.4 in stage II/III, *p*‐value = 0.921). Cytokines play an important role in inducing macrophage polarization during the macrophage transition. Th1 cytokines such as interleukin‐12 (IL‐12) and IL‐18 or activated Toll‐like receptors (TLRs) can promote macrophages to M1 polarization, while Th2 cytokines such as IL‐4, IL‐10, and IL‐13 induce M2 polarization.^33^ In addition, tumors can elicit hypoxia and cell death to induce the tumor‐promoting reprogramming of macrophages.^29^ The detailed mechanisms underlying the TAM pattern switch need further investigation. Meanwhile, identifying TAM pattern change holds promise for TAM‐focused therapeutic strategies, such as re‐educating TAMs to the protumoral, M1‐like status.^31^ A wealth of approaches, such as radiotherapy and chemotherapeutics, have targeted TAM phenotype and polarization to promote the antitumoral M1 TAMs, and it is critical to ensure that the activation of M1 phenotype can lead to tumor‐antagonizing outcomes.^28^ Additionally, we checked the relationships between TAMs and immunotherapy‐related biomarkers. A recent study suggested PD‐L1 high expression is associated with increased stromal M2 TAM density.^34^ Probably due to the very early stage of NSCLC in our research, we did not observe any apparent correlation between TAM composition and driver mutation type, TMB, or PD‐L1 level.

We also assessed tumor‐infiltrating CD8+ T cells and NK cells in the TME. Strong infiltration of CD8+ T cells is usually associated with a better prognosis.^35^ However, the trafficking and infiltration of T cells may be hampered by the TME of solid tumors, and hypoxia in lung TME could cause a harsh environment to limit their survival and effector functions.^36^ We found relatively low infiltration of CD8+ T cells in tumor parenchyma of stage IA NSCLC, but several markers, including age < 50, driver mutations in *RAS‐MAPK* pathway, and TMB‐H, appeared to be associated with relatively higher CD8+ T cell filtration. Previous studies have also found a correlation between *KRAS* mutations and increased CD8+ T cell infiltration in the TME, which is accompanied by elevated intratumoral PD‐L1 expression.^37^, ^38^ We observed moderately increased infiltration of CD8+ T cells in PD‐L1‐H tumor tissues but this lacked statistical significance. Our NGS analysis also generated helpful information. Over half of the patients in our study carried *EGFR* driver mutations, and they showed relatively lower CD8+ T cell infiltration than the *RAS‐MAPK* mutation patients in tumor parenchyma (Figure 3B). T cell apoptosis induced by *EGFR* mutations can contribute to the lower density of infiltrating CD8+ lymphocytes in *EGFR* mutants.^39^ Inhibiting CD8+ T cell apoptosis may benefit NSCLC patients carrying *EGFR* mutations for their responsiveness to immunotherapy. The TMB‐H samples significantly increased infiltrating CD8+ T cells in tumor parenchyma (Figure 4A). High TMB tumors can yield more immunogenic neoantigens to be recognized by the immune system, such as CD8+ effector T cells.^40^, ^41^ A previous study suggested that a high mutation burden is associated with increased tumor infiltration by activated T cells in the case of lung squamous cell carcinoma.^42^ It would further be of interest to dissect neoepitope‐specific CD8+ T cells and understand the relationships between TMB and T cell functionality in NSCLC. The immune contexture, particularly the T cell landscape, has been shown to evolve across different stages of early lung cancer pathogenesis. Using immune gene expression profiling data, Dejima et al. evaluated the changes in immune cell composition and revealed that the fraction of CD8+ T cells increased from MIA to ADC,^43^ consistent with our discovery by mIHC (Figure S3).

Infiltration of NK cells represents a favorable prognosis,^44^ and we observed a higher density of total infiltrating NK cells in tumor parenchyma of stage IA NSCLC (Figure S1B). Most NK cells in the lung show the CD56dim phenotype,^45^ consistent with the dominance of the CD56dim subpopulation we observed. Functionally, the highly differentiated CD56dim NK cells can express killer cell immunoglobulin‐like receptor (KIR) with potent cytotoxicity and confer cytolytic activity, while the less mature CD56bright NK cells often lack KIR expression but are the major cytokine producer.^46^ The expression of PD‐L1 can create an immunosuppressive microenvironment that prevents NK cell‐mediated lysis.^47^ We observed a relative decrease of the cytolytic CD56dim NK cells and an increased proportion of the cytokine‐secreting CD56bright cells in PD‐L1 high samples (Figure 5A). It would be informative to check if blocking PD‐L1 could promote CD56bright cell differentiation into CD56dim cells.^48^

We acknowledge the limitations of this study. We carried out mIHC to characterize the stage IA NSCLC TME, but this technique can only detect limited markers simultaneously. Thus, in the following studies, we plan to focus our research and dissect the subpopulations with higher resolution, such as the naïve and differentiated states in the CD8+ T cell compartment. Meanwhile, our findings can be justified by other single‐cell approaches, such as mass cytometry, single‐cell RNA‐seq, spatial transcriptomics, and systems biology. As the TME is a multidimensional network, combinatorial techniques can assist in understanding the crosstalk between immune and nonmalignant cells. The relatively small cohort size also affected the interpretation of the results, and we plan to expand this study in the future. For instance, only two patients were in the high TMB cohort, and a more accurate estimate would require the analysis of larger cohorts. Enlarging the cohort would also allow us to further assess the stepwise transition of immune cell infiltration during cancer development and the effects of specific mutations. Furthermore, due to the lack of postoperative information, incorporation of prognostic outcomes and response to treatment would be particularly informative in future studies.

Using the representatives of CD8+ T cells, TAMs, and NK cells, we profiled the spatial distribution of infiltrating immune cells and depicted a heterogeneous TME landscape for stage IA NSCLC. The early‐stage TME demonstrated a higher proportion of antitumoral M1 polarization in the TAM compartment. We also found age, driver mutation type, PD‐L1, and TMB levels could contribute to the heterogeneity of infiltrating immune cells in the TME. Overall, our findings shed light on understanding the TME of early‐stage NSCLC and potentially promoting immunotherapy.

## CONFLICT OF INTEREST

XC, Yang Shao, and YL are employees of Nanjing Geneseeq Technology Inc., China. All other authors have declared no conflicts of interest.

## Supporting information


**Appendix** **S1: Supporting information**
**Supporting Table S1.** Clinical characteristics of the stage II and III lung cancer patients.
**Supporting Figure S1.** Spatial density of total (A) TAMs, and (B) NK cells in different tissue samples of the 33 stage IA patient samples (TAM: tumor‐associated macrophage; NK cells: natural killer cells; TP: tumor parenchyma; TS: tumor stroma; PA: paracancerous tissue; NT: nontumor tissue).
**Supporting Figure S2.** Abundance of (A) TAMs, (B) CD8+ T cells, and (C) NK cells in tumor parenchyma between stage I and stage II/III patients (TAM: tumor‐associated macrophage; NK cells: natural killer cells).
**Supporting Figure S3.** Abundance of (A) CD8+ T cells, (B) TAMs, and (C) NK cells in tumor parenchyma and stroma between MIA and ADC patients (TAM: tumor‐associated macrophage; NK cells: natural killer cells; MIA: minimally invasive adenocarcinoma; ADC: invasive adenocarcinoma).
**Supporting Figure S4.** Summary of mutations in the 33 stage IA NSCLC patients (CNV: copy number variation; SV: structural variation).
**Supporting Figure S5.** Abundance of (A) CD8+ T cells, (B) TAMs, and (C) NK cells in tumor parenchyma and stroma between *TP53* mutated (TP53+) and *wildtype* (TP53‐) patients (TAM: tumor‐associated macrophage; NK cells: natural killer cells).
