# Supplementary material for: Multidimensional profiling depicts infiltrating immune cell heterogeneity in the tumor microenvironment of stage IA non‐small cell lung cancer
Source: Thorac Cancer. 2022 Feb 11;13(7):947–55. doi: 10.1111/1759-7714.14329 (PMC8977165; doi:10.1111/1759-7714.14329)

**Supporting Table S1.** Clinical characteristics of the stage II and III lung cancer patients.

| **Characteristics** | **All Patients (*N*=5)** |
| --- | --- |
| **Median age, years (range)** | 49 (43-56) |
| **Median tumor diameter, cm (range)** | 2.5 (2.0-5.0) |
| **Sex, *n* (%)** |  |
| Male | 1 (20.0%) |
| Female | 4 (80.0%) |
| **Histology, *n* (%)** |  |
| ADC † | 5 (100.0%) |
| **Stage, *n* (%)** |  |
| IIA | 2 (40.0%) |
| IIB | 2 (40.0%) |
| IIIA | 1 (20.0%) |
| **TNM, *n* (%)** |  |
| T1c | 1 (20.0%) |
| T2 | 3 (60.0%) |
| T3 | 1 (20.0%) |
| **Smoking, *n* (%)** |  |
| Current Smoker or Ever Smoker | 1 (20.0%) |
| Never smoker | 4 (80.0%) |
|  |  |

†ADC: Invasive adenocarcinoma

**Supporting Figure S1.** Spatial density of total (A) TAMs, and (B) NK cells in different tissue samples of the 33 stage IA patient samples (TAM: tumor-associated macrophage; NK cells: natural killer cells; TP: tumor parenchyma; TS: tumor stroma; PA: paracancerous tissue; NT: nontumor tissue).


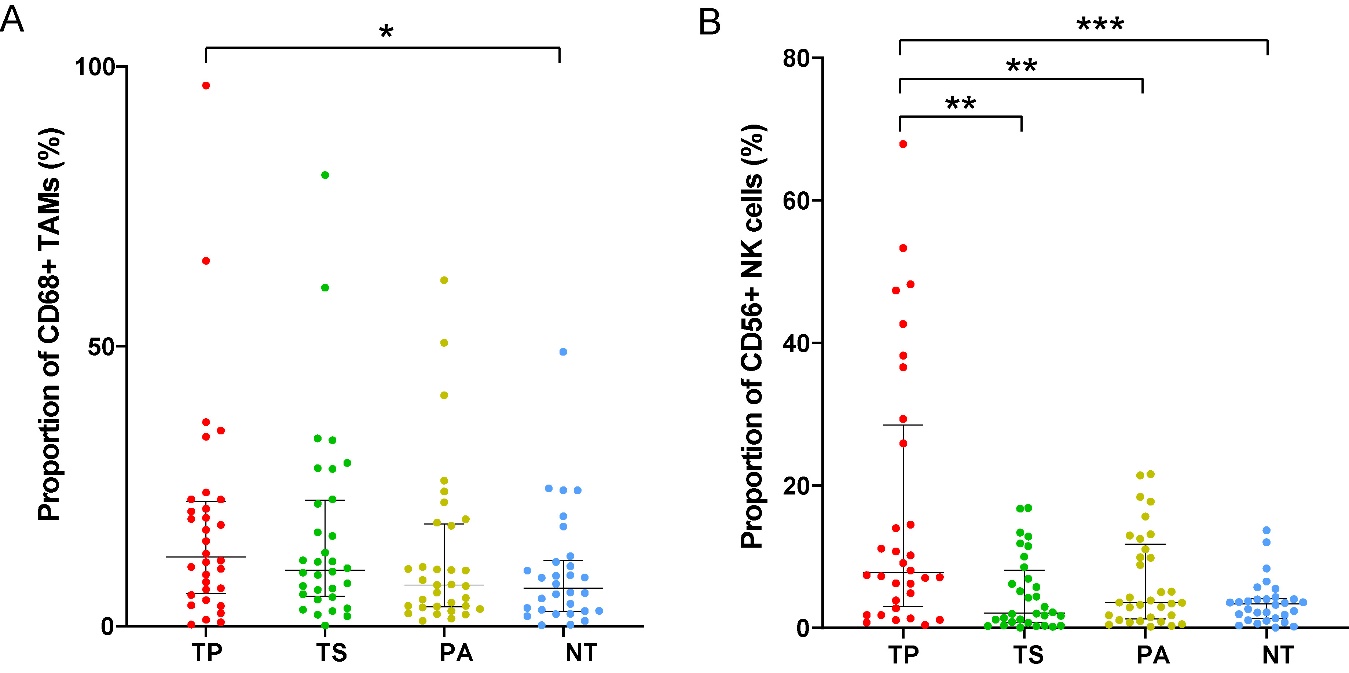


**Supporting Figure S2.** Abundance of (A) TAMs, (B) CD8+ T cells, and (C) NK cells in tumor parenchyma between stage I and stage II/III patients (TAM: tumor-associated macrophage; NK cells: natural killer cells).


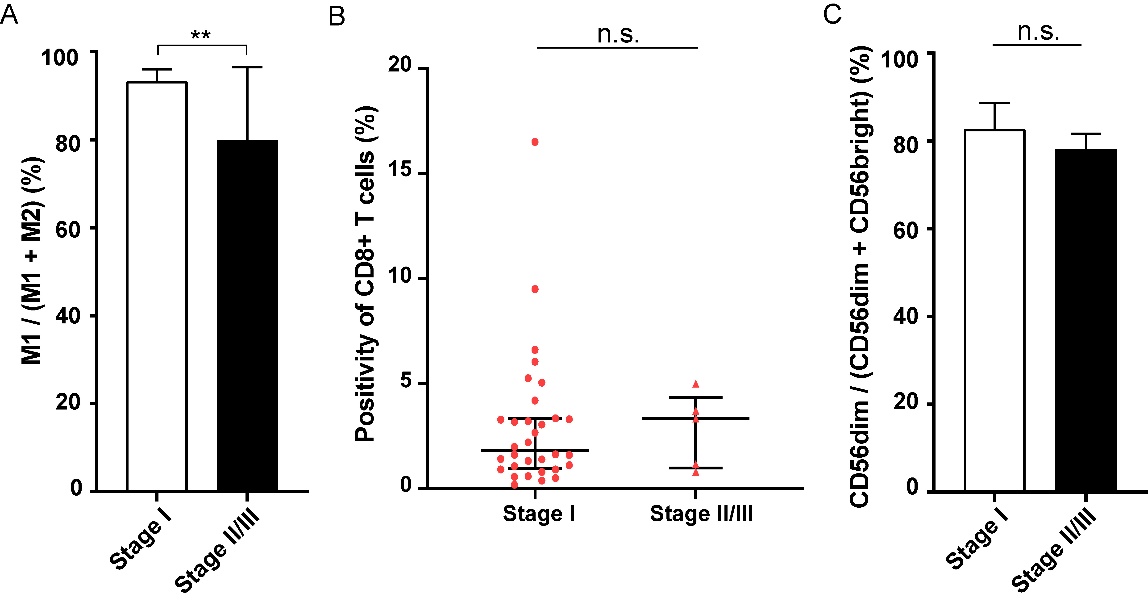


**Supporting Figure S3.** Abundance of (A) CD8+ T cells, (B) TAMs, and (C) NK cells in tumor parenchyma and stroma between MIA and ADC patients (TAM: tumor-associated macrophage; NK cells: natural killer cells; MIA: minimally invasive adenocarcinoma; ADC: invasive adenocarcinoma).


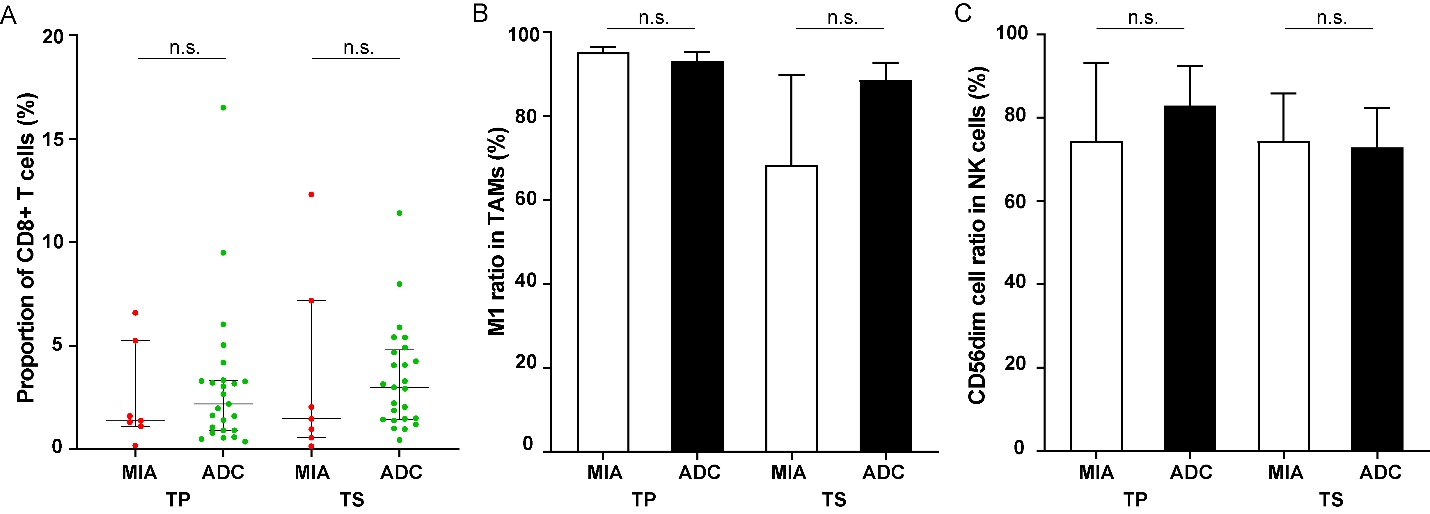


**Supporting Figure S4.** Summary of mutations in the 33 stage IA NSCLC patients (CNV: copy number variation; SV: structural variation).


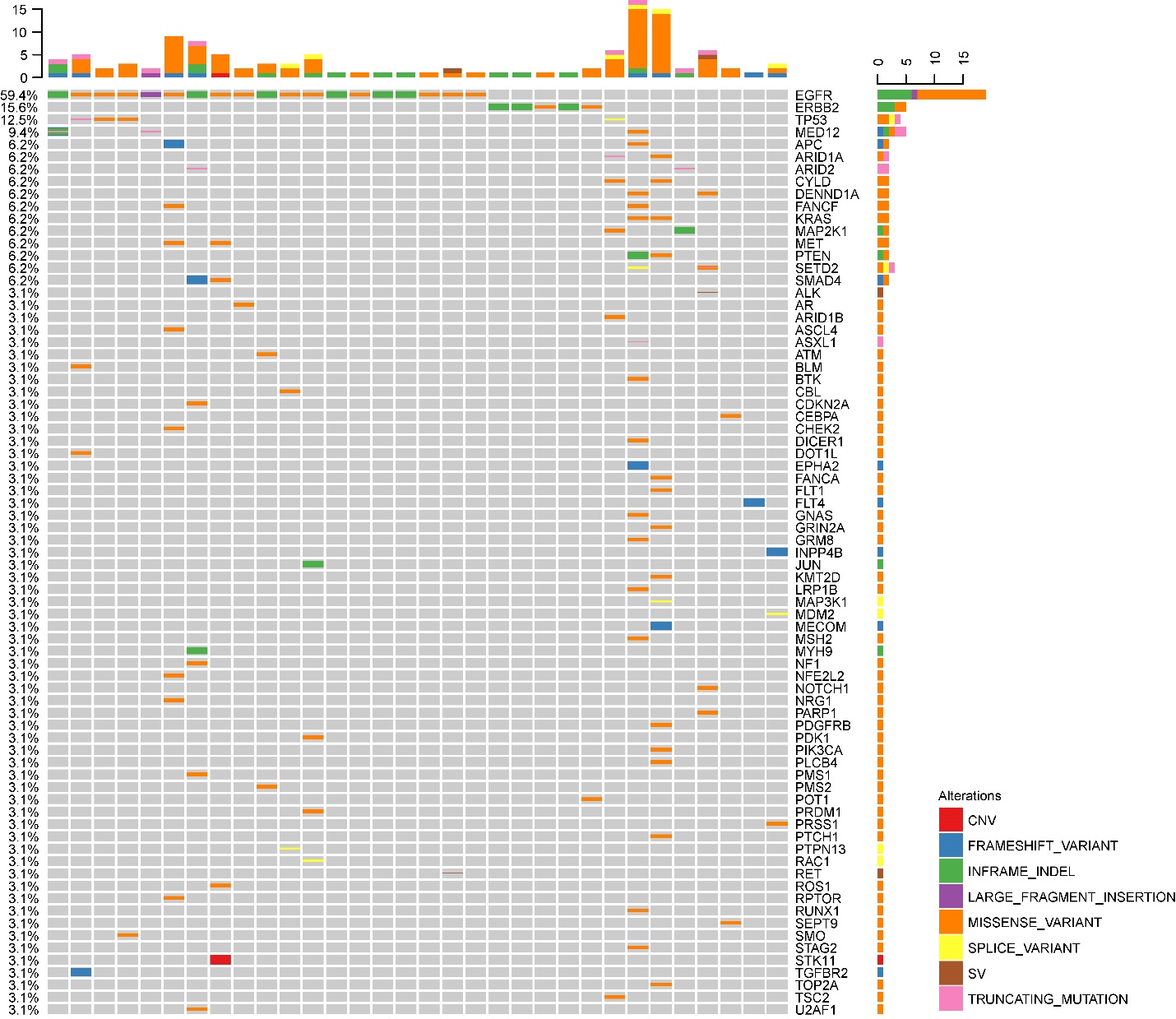


**Supporting Figure S5.** Abundance of (A) CD8+ T cells, (B) TAMs, and (C) NK cells in tumor parenchyma and stroma between *TP53* mutated (TP53+) and *wildtype* (TP53-) patients (TAM: tumor-associated macrophage; NK cells: natural killer cells).


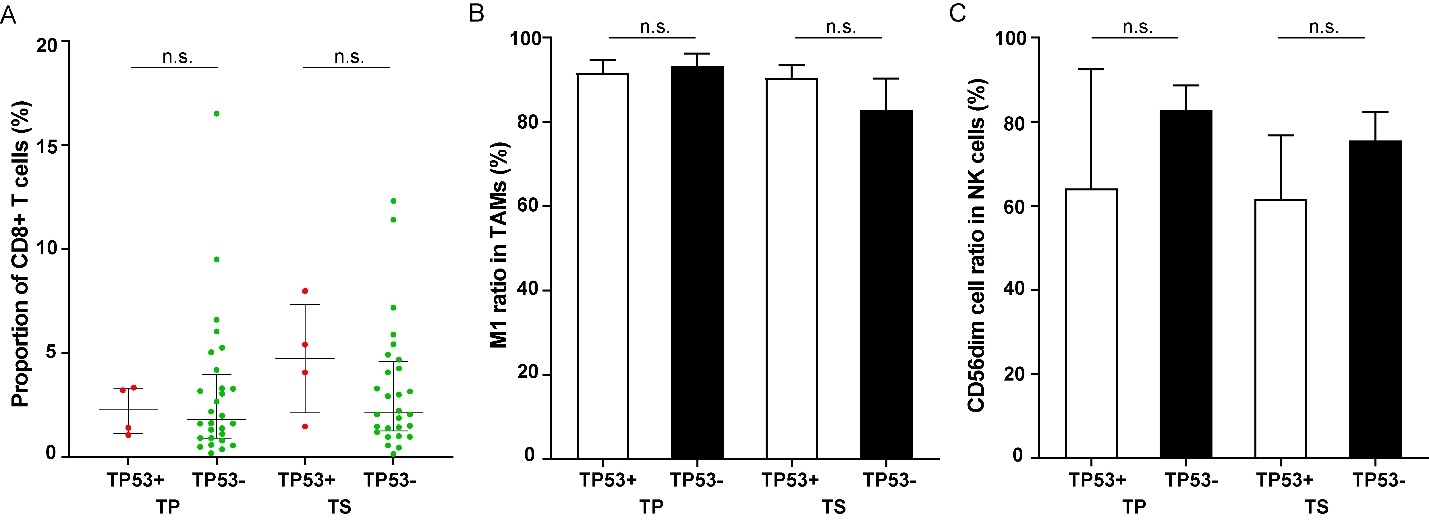

Supplement: Supplementary file 1 — Appendix S1: Supporting information Supporting Table S1. Clinical characteristics of the stage II and III lung cancer patients. Supporting Figure S1. Spatial density of total (A) TAMs, and (B) NK cells in different tissue samples of the 33 stage IA patient samples (TAM: tumor‐associated macrophage; NK cells: natural killer cells; TP: tumor parenchyma; TS: tumor stroma; PA: paracancerous tissue; NT: nontumor tissue). Supporting Figure S2. Abundance of (A) TAMs, (B) CD8+ T cells, and (C) NK cells in tumor parenchyma between stage I and stage II/III patients (TAM: tumor‐associated macrophage; NK cells: natural killer cells). Supporting Figure S3. Abundance of (A) CD8+ T cells, (B) TAMs, and (C) NK cells in tumor parenchyma and stroma between MIA and ADC patients (TAM: tumor‐associated macrophage; NK cells: natural killer cells; MIA: minimally invasive adenocarcinoma; ADC: invasive adenocarcinoma). Supporting Figure S4. Summary of mutations in the 33 stage IA NSCLC patients (CNV: copy number variation; SV: structural variation). Supporting Figure S5. Abundance of (A) CD8+ T cells, (B) TAMs, and (C) NK cells in tumor parenchyma and stroma between TP53 mutated (TP53+) and wildtype (TP53‐) patients (TAM: tumor‐associated macrophage; NK cells: natural killer cells). [file TCA-13-947-s001.docx]
